# Solid-state dewetting templates the formation of an atomically dispersed gold phase†

**DOI:** 10.1039/d6na00466k

**Published:** 2026-06-25

**Authors:** Ravalika Sajja, Marcos V. S. Martins, Rongsheng Cai, Abdulghani Ismail, Gwang-Hyeon Nam, Ylea Vlamidis, Neeraj Mishra, Max Rimmer, Stefan Heun, Camilla Coletti, Mark A. Isaacs, Ashok Keerthi, Sarah J. Haigh, Stefano Veronesi, Boya Radha

**Affiliations:** a Department of Physics & Astronomy, The University of Manchester Oxford Road Manchester M13 9PL UK radha.boya@manchester.ac.uk; b National Graphene Institute, The University of Manchester Oxford Road Manchester M13 9PL UK; c Department of Materials, The University of Manchester Oxford Road Manchester M13 9PL UK; d Istituto Nanoscienze – CNR, NEST – Scuola Normale Superiore Piazza S. Silvestro 12 56127 Pisa Italy; e Department of Physical Science, Earth, and Environment, University of Siena Via Roma 56 53100 Siena Italy; f Center for Nanotechnology Innovation @ NEST, Istituto Italiano di Tecnologia Piazza S. Silvestro 12 56127 Pisa Italy; g Graphene Labs, Istituto Italiano di Tecnologia Via Morego 30 Genova 16163 Italy; h Department of Chemistry, University College London 20 Gordon Street London WC1H 0AJ UK; i HarwellXPS, Research Complex at Harwell, Rutherford Appleton Labs Harwell Campus OX11 0FA UK; j Department of Chemistry, The University of Manchester Oxford Road Manchester M13 9PL UK

## Abstract

We report the formation of a stable phase of gold single atoms driven by solid-state dewetting of nanoplates. Through atomically resolved approaches and combined spectroscopy, our study provides a still unexplored perspective on the solid–solid interface of gold crystals undergoing dewetting, unveiling its capability to stabilise exposed atomically dispersed phases as dense as ∼20 single atoms per 100 nm^2^.

## Abstract 2

When undergoing solid-state dewetting, the active solid–solid interface of gold nanoplates drives the formation of a stable atomically dispersed phase.

## Graphical abstract

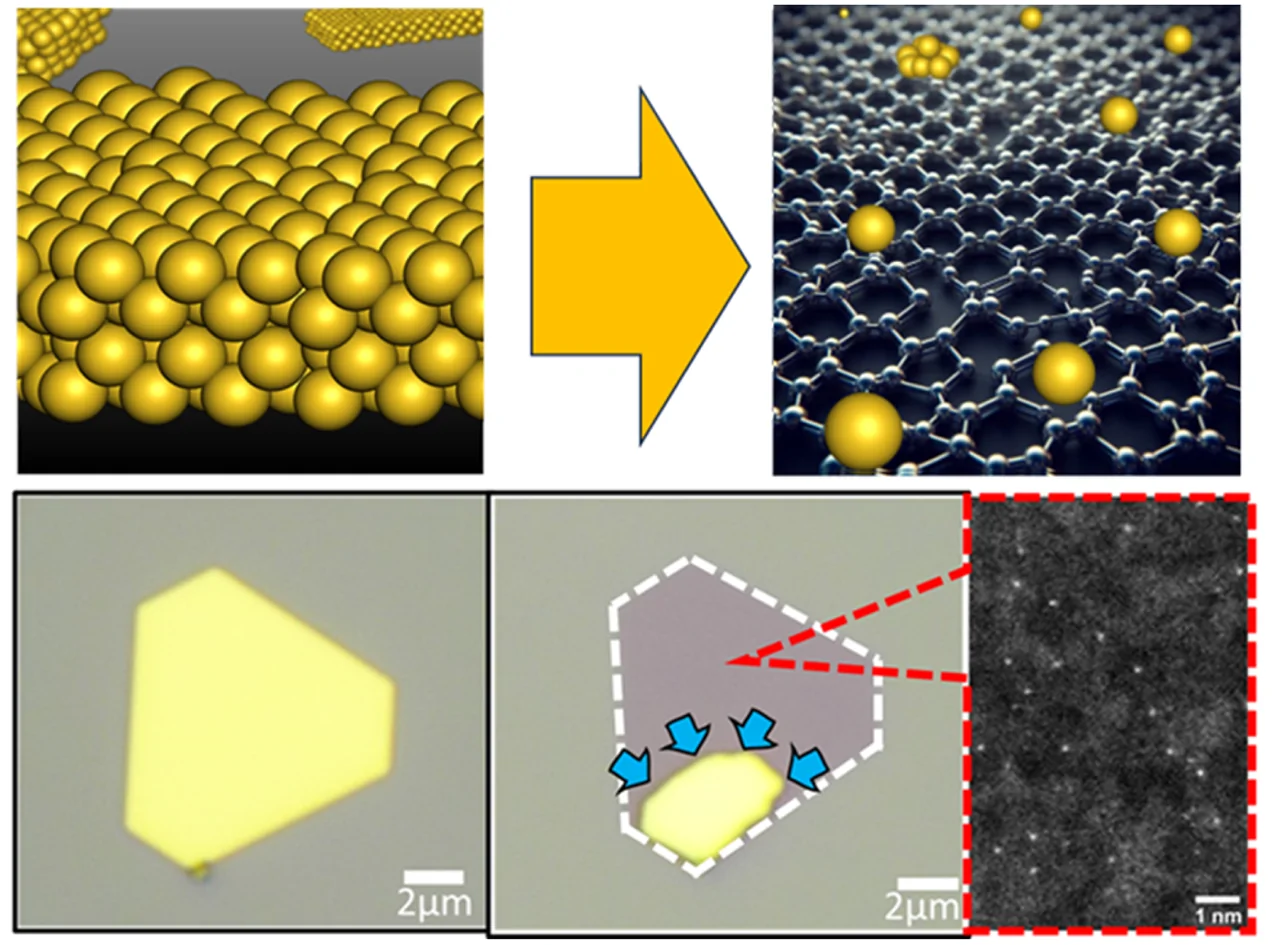

Solid state dewetting (SSD) has been at the intersection of opposing frontlines in materials development over recent years. On one side, it underpins a critical effect challenging post-processing stages of metallic thin films and microcircuits,^1^ whereas on the other it has emerged as an opportunity for advancements in nanoparticle production.^2–4^ For the latter, however, continuous efforts still drive recent studies since the mechanism leads to a rather broad distribution of particle sizes. Briefly, SSD is a spontaneous process by which thin solid films or crystals rearrange into smaller particles at temperatures far below the melting point, essentially driven by surface energy minimisation.^5,6^ As a dynamic process, adatoms travel between coalescing particles as these grow at the expense of their neighbours, thus securing a non-uniform array of particles. SSD has been extensively investigated for a wide range of metals and alloys. The tendency of a thin film or crystal towards dewetting depends on a series of parameters such as its thickness, temperature gradients across the surface, and adatom diffusivity.^5^ Although several experimental studies show the structural evolution of single crystals or grains during transformation,^1–4,7–10^ a limited number provide the active mechanism at the metal/substrate interface and surface effects on the stability of such dispersed adatom species. A systematic view on the latter could provide a deeper understanding and potentially new strategies to mitigate the poor stability of atomically dispersed (AD) materials.

Atomically dispersed metals (ADMs) display distinct chemical and physical attributes with potential uses^11–14^ in high-performance catalysts,^15^ in selective gas/bio sensors,^16–18^ and as efficient nanozymes.^17,19^ In the AD phase, the low dimensionality maximises utilization of each individual active site in contrast to bulk materials where only the surface atoms or those at defect sites are available.^20,21^ Over the past two decades, efforts have been made in both synthesis^22–24^ and characterization^25^ of ADMs.^20^ Most of the challenges brought along with such new methods revolve around the control and stabilisation of the resulting ADMs such as composition, the level of dispersion, and valence states.^26^ The fabrication of ADMs is challenging due to the tendency of isolated metal atoms to aggregate both during synthesis and during any subsequent treatments^27^ due to the high free energy associated with AD species. Both solution and solid-state methods have been harnessed to create ADMs.^28^ Solid state methods or dry techniques have no solvents and can be advantageous for specific high-temperature reactions. Nonetheless, approaches such as atomic layer deposition, pyrolysis, ball milling, and atom trapping^28^ bring limited control over the coordination environment of the synthesised metal atoms. Overall, several challenges persist in this research domain. For instance, the bonding instability arising from the high free energy of individual metal atoms and stability against migration, diffusion, and aggregation pose long-standing challenges in the creation of stable ADMs.

In this work, we use gold (Au) single crystals for the formation of an atomically dispersed Au (AD-Au) phase upon solid state dewetting. We then show that stable Au single atoms are stabilised along with a carbon-based supporting layer. This self-assembling layer is inherent to the process and acts as an interfacial buffer which is independent of the composition or geometry of the material used as the substrate. We use a multidisciplinary characterisation process to shed light on the phenomenon underlying the formation and stabilisation of such an AD phase. Our findings suggest that dewetting of the intermediate Au nanoplates triggers the formation of AD-Au distributed across the carbon-based supporting layer.

Synthesis of the AD-Au phase is schematically shown in Fig. 1a. Initially, we grew Au platelets on a Si/SiO_2_ substrate based on previous reports.^29,30^ Briefly, we carry out thermolysis of the gold complex to form Au nanoplates. Initially we have an aqueous phase of hydrogen tetrachloroaurate (HAuCl_4_), combined with phase transfer agent, tetraoctylammonium bromide (ToABr) in toluene, and Au combines with ToABr into a metal–organic complex in the organic phase. Upon drop-casting this complex onto the substrate of choice and thermolyzing, the organic component acts as a medium where Au crystallizes upon thermal reduction to form Au nanoplates. The as-formed quasi-2-dimensional Au nanoplates exhibit hexagonal, triangular, and truncated triangular shapes with edge lengths ranging from ∼5 to 20 µm (see Fig. 1b and S1 for lower magnification). Atomic force microscopy of a large population of crystals reveals that the population of Au nanoplates is formed with thicknesses ranging between 32 nm and 120 nm. We further carry out an annealing step at 750 °C under an Ar/H_2_ (10%) atmosphere to both pyrolyze the hydrocarbon content from the remaining organic phase transfer agent and trigger the formation of the AD-Au phase for 12 hours. During the latter, as the nanoplates coalesce into a few micrometre-sized bulk gold domains, it leaves behind a uniformly distributed population of Au single atoms spread across the carbonaceous surface layer (Fig. 1c). From optimisation experiments, we observe that such phase transformation generating the population of Au single atoms remains continuously reproducible up to annealing temperatures of 800 °C under the same Ar/H_2_ atmosphere (Fig. 1d). As shown in Fig. 1b–d, the carbonaceous layer supporting the AD-Au phase perfectly retains the contours of the initial gold nanoplates.^31^ We analysed the influence of the substrate, by performing the synthesis on quartz and epitaxial graphene on silicon carbide. Overall, the whole process shows good reproducibility and does not discriminate the substrate's structure or composition thanks to the carbonaceous supporting layer, which acts as a buffer stabilising any interfacial incompatibility between the Au phases and the substrate (SI, Fig. S2). This intrinsic feature is what sustains the versatility of the method as this carbon-based layer originated from the organic phase transfer agent used in early stages.

**Fig. 1:**
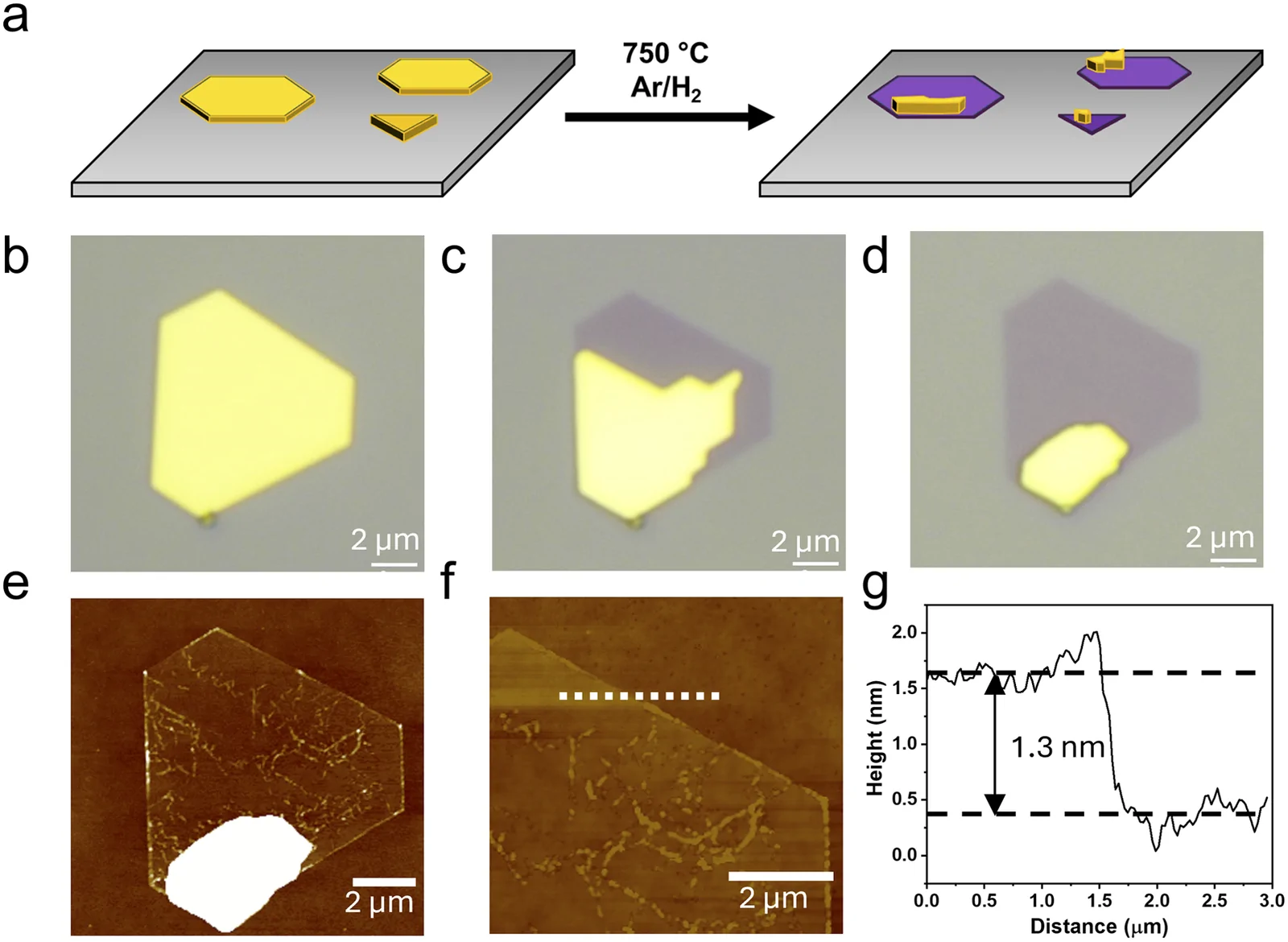
Synthesising atomically dispersed gold (AD-Au). (a) Schematic representation outlining the strategy for creation of AD-Au from Au nanoplates. (b–d) Optical images illustrating the evolution of AD-Au originating from an Au nanoplate (b) under an Ar/H_2_ atmosphere upon 12 hours of annealing at 750 °C (c) and further 11 hours of annealing at 800 °C (d) and (e) AFM image of the nanoplate encompassing the aggregated bulk gold region and the AD-Au region. (f) Magnified AFM image featuring only the AD-Au. (g) The AD-Au region of the plate thickness is ∼1.3 nm, as shown in the AFM height profile.

To understand the role of the atmosphere, we performed pyrolysis using multiple carrier gases and a vacuum. While we notice that in a vacuum only part of the supporting layer is maintained, the use of the oxidising atmosphere turned out to be catastrophic as it triggers the combustion of the carbonaceous layer and potential volatilisation of the AD-Au phase (SI, Fig. S3). Therefore, a chemically reducing or inert atmosphere is crucial for the development of the AD-Au phase as it does not affect the structure and stability of the carbonaceous layer supporting it. Previous studies have shown that the presence of hydrogen gas in thermal–reduction reactions of carbon- and graphene-based materials leads to the regeneration and/or formation of graphitic sp^2^ domains.^14^ Moreover, the same H_2_-promoted conversion is known for graphitisation reactions from aliphatic reactants.^32^ Thus, we propose that graphitisation also occurs in our system to a certain extent, securing the formation of a robust and chemically stable support layer. Indeed, as also presented in Fig. S3, a more robust and continuous supporting layer was formed for the samples treated under an Ar-10% H_2_ atmosphere compared with that formed under pure Ar. The average thickness of the carbonaceous layer is consistent across batches and falls around 1.3 nm (Fig. 1g) After the annealing stage (Fig. 1d), the coalesced bulk Au phase became 270 nm thick (Fig. S4), roughly 3–8 times larger than the original nanoplate, initially 35 nm thick. Such rearrangement is likely driven by the high surface free energy of the metallic phase that can be minimised by turning the platelet into a more isotropic volume, with less surface area.^33,34^

To characterise the AD-Au, we used aberration corrected high-angle annular dark field-scanning transmission electron microscopy (HAADF-STEM). To prepare our samples, we used the well-established protocol for lifting and transferring low-dimensional materials across different substrates.^35^ This approach is widely used in nanofabrication routines using nanomaterials in general and it basically consists of detaching the nanoplates and the supporting carbonaceous layer from the substrate by selectively etching the SiO_2_ passivation layer of the substrate with a strong base. The nanostructures are then transferred onto a holey carbon transmission electron microscopy (TEM) support grid. Interestingly, all the coalesced bulk Au, AD-Au phase, and the carbonaceous supporting layer are robust enough to be transferred as a whole, maintaining their integrity and relative positions during the transfer process. Fig. 2a displays the sample on the TEM grid, whereas Fig. 2b provides a magnified view of the area enclosed by a yellow rectangular box in panel a. As the atomic number of the gold is much higher than that of any element forming the supporting layer, it appears bright in the *Z*-contrast of the HAADF STEM imaging mode. Examination of the region shown in Fig. 2b at higher magnification reveals the presence of AD-Au atoms on the support (Fig. 2c–e, SI, Fig. S8). Energy dispersive X-ray spectroscopy (EDS) of the areas along the carbonaceous support shown in Fig. 2g confirms the absence of any high atomic number species other than Au and Cu, the latter expected from the TEM support grid and polepiece (SI, Fig. S5a).^36^ The AD-Au phase is uniformly distributed (Fig. 2b–e and S5b–d), with a highly reproducible average density of Au atoms of ∼20 atoms per 100 nm^2^.

**Fig. 2:**
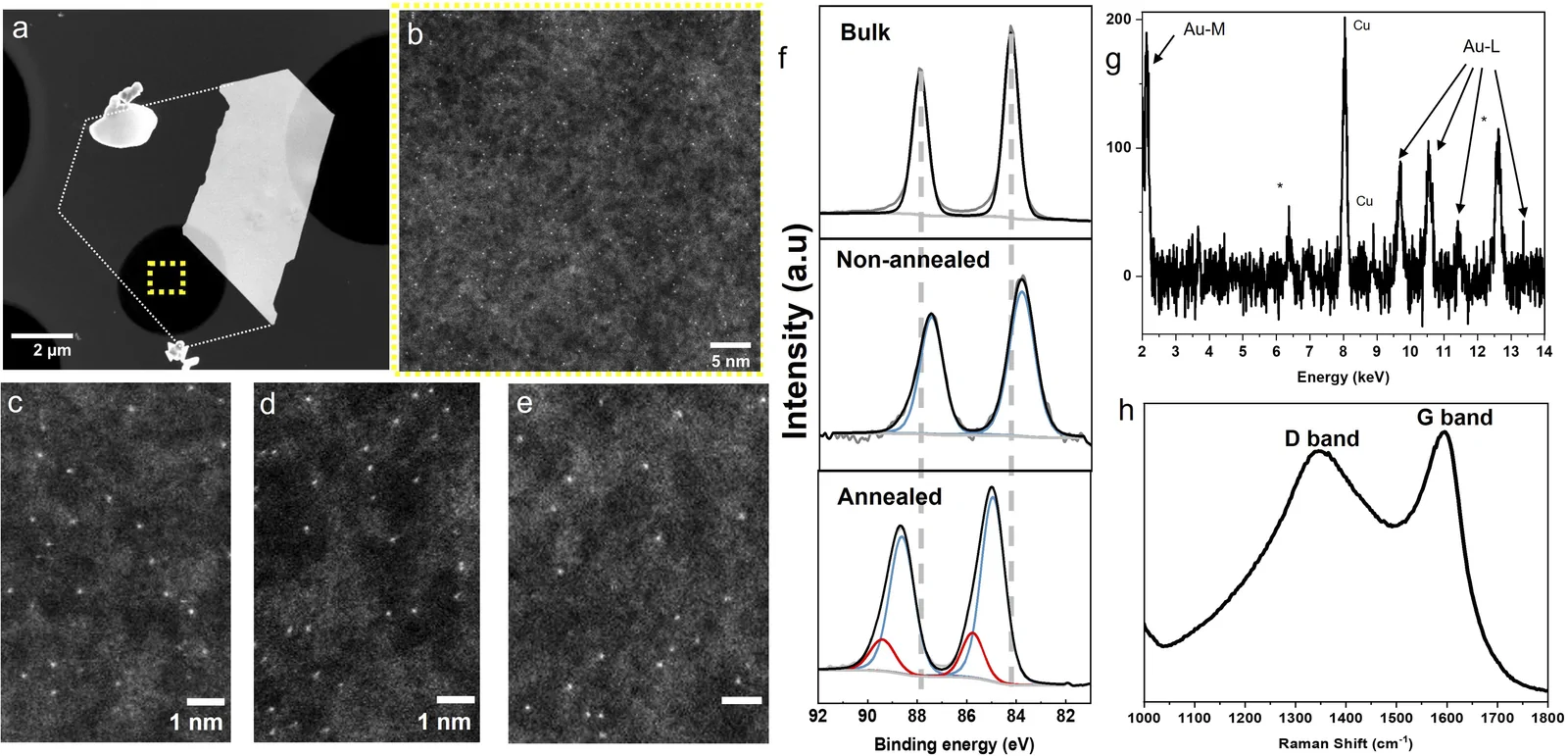
Characterisation of the AD-Au region. (a) HAADF STEM image of a pyrolyzed gold nanoplate on a TEM grid. Area of the carbon support is indicated by a white dashed line. The brightest feature is the agglomerated bulk Au region. (b) HAADF-STEM image of the AD-Au region corresponding to the area highlighted by a yellow rectangle in panel (a). (c–e) Higher magnification HAADF-STEM images of the AD-Au in the region. (f) Average Au 4f XPS spectra obtained from an AD-Au sample (bottom), a non-annealed Au nanoplate (middle) and the bulk (top). The 4f peaks from the bulk are displayed in blue, while additional peaks contributing to the peak width are shown in red. The peak position comparison already accounts for charge correction using a graphite flake as a reference and Shirley background; (g) STEM EDS spectrum obtained from the sample area depicted by the yellow square in panel (a) indicating the presence of Au – additional peaks include copper (8.04 and 8.90 keV), which originated from the TEM support grid and the peaks marked by * (6.5 keV and 12.5 keV) arise from instrumental sources. (h) Raman spectrum from the AD-Au region indicating the presence of a carbonaceous matrix.

The bonding coordination of Au was analysed using X-ray photoelectron spectroscopy (XPS) performed on the Si/SiO_2_ substrates for samples before and after annealing along with a comparison to reference bulk Au (200 nm thick continuous Au film, Fig. 2f). The characteristic Au 4f lines were observed around a nominal binding energy of 84.5 eV and 88 eV for 4f_7/2_ and 4f_5/2_, respectively. In comparison with the bulk reference, a blue-shift of ∼0.5 to 0.7 eV was observed for the AD-Au samples along with a significant broadening in the Au 4f_5/2_ and Au 4f_7/2_ peaks (bottom panel of Fig. 2f). A similar XPS peak shift was reported for Pt nanoclusters and single atoms on carbon-based materials.^37–39^ Considering the quantum effects, we cannot rule out the possibility of these shifts being predominantly influenced by reduced size or dimensionality of our Au as well as forming binding structures with the reduced carbonaceous species in the substrate,^40–43^ or even silicides formed along disruptions of SiO_2_ passivation layer caused by the reducing atmosphere treatment at higher temperatures.^44^ Conversely, as the as-grown nanoplates still possess a significant amount of non-graphitised hydrocarbons from the precursors, we attribute the red-shifts observed in their Au 4f spectra (middle panel of Fig. 2f) to the influence of such species on the structure, together with induced charging effects. Overall, the relatively consistent peak positions among several samples indicate that the bonding of the Au remaining on the carbonaceous layer after annealing differs significantly compared to bulk Au. In addition, the use of the Auger parameter has been shown in the recent literature as an efficient and straightforward way to distinguish the oxidation state of transition and noble metals.^45^ While standard Al Kα lab sources can access the Au NOO transition, this possesses low kinetic energy (∼240 eV kinetic energy) and therefore becomes difficult to analyse, since it becomes swamped in the low energy secondary-electron background signal. Using a higher energy source, Ag Lα, we can instead access the Au MNN Auger–Meitner peak (∼2100 eV kinetic energy) – which presents a much stronger and more well-defined structure. When we consider the Au 4f – Au MNN Auger parameter, we observe reduced screening of Au atoms potentially due to the transition from bulk metallic like to more isolated atom character with fewer polarisable electrons capable of screening the core-hole. The data are summarised in Fig. S6. This additional approach corroborates well with the shifts observed with the Al source.

We used Raman spectroscopy to assess the amorphous-to-graphitic carbon ratio in the carbonaceous supporting layer (Fig. 2h). A blue-shifted and broadened G band at 1590 cm^−1^ is observed along with a disorder-induced D band at 1345 cm^−1^. This contrasts with graphene, a highly crystalline carbon which typically displays a single sharp peak at around 1580 cm^−1^. For our carbon layer, the average *I*_D_/*I*_G_ ratio is close to 1, indicating a highly distorted and amorphous structure. Similar *I*_D_/I_G_ ratios have been observed in previous Raman studies of materials like graphene oxide^46^ or amorphous carbon.^47^ The disordered nature of the carbon film is further confirmed by the diffuse-scattering ring under electron diffraction (Fig. S7) and the absence of long-range crystallinity visible by TEM imaging, as well as by the XPS C 1s lines (SI, Fig. S8), which indicates C-sp^3^ as its main constituent.

To further distinguish whether the AD-Au is surface-bound or embedded into the carbonaceous matrix of the supporting layer, we performed direct imaging *via* scanning tunnelling microscopy (STM). The samples for STM were prepared on conductive epitaxial graphene on a 6H-SiC (0001) substrate. We used point-mode scanning tunnelling spectroscopy (STS) to discern the electronic structure of both Au-AD domains and their surroundings. The differential conductivity (d*I*/d*V*) plotted against the sample bias voltage (*V*) acquired from the AD-Au region reveals a semiconducting feature. Conversely, the expected semi-metallic behaviour of graphene is evident in the curves obtained from the epitaxial graphene areas (Fig. S9f), whereas the agglomerated bulk gold holds a distinct metallic behaviour (Fig. S9d).

## Insights into the AD-Au formation mechanism on an amorphous carbon support

The temperature used in the pyrolytic reaction is not only essential to trigger the formation of the carbonaceous layer but also to activate the structural transitions observed in the metallic phase. For the latter, solid-state dewetting is likely to define the mechanism of these transformations. This phenomenon taking place below the melting temperature has been vastly studied.^5^ At sufficiently high temperatures still below the melting point, surface diffusion takes place to minimise the surface energy, especially when the crystal is very thin. The mobility acquired by adatoms is known to underlie the Ostwald ripening effects in similar systems where atoms travel along the surface between neighbouring structures thus allowing one to grow at the expense of the other. In our case, however, the mobility of diffusing atoms is likely limited by both the rough terrain provided by the carbonaceous layer and the Ehrlich–Schwoebel barrier rising between the step-edge of the carbonaceous layer and SiO_2_. In such regions, the considerably large populations of adatoms are commonly repelled due to the high energy penalty thus becoming restricted to the carbonaceous layer. This effect explains the notably high density of single atoms observed along the carbonaceous layer.

**Fig. 3:**
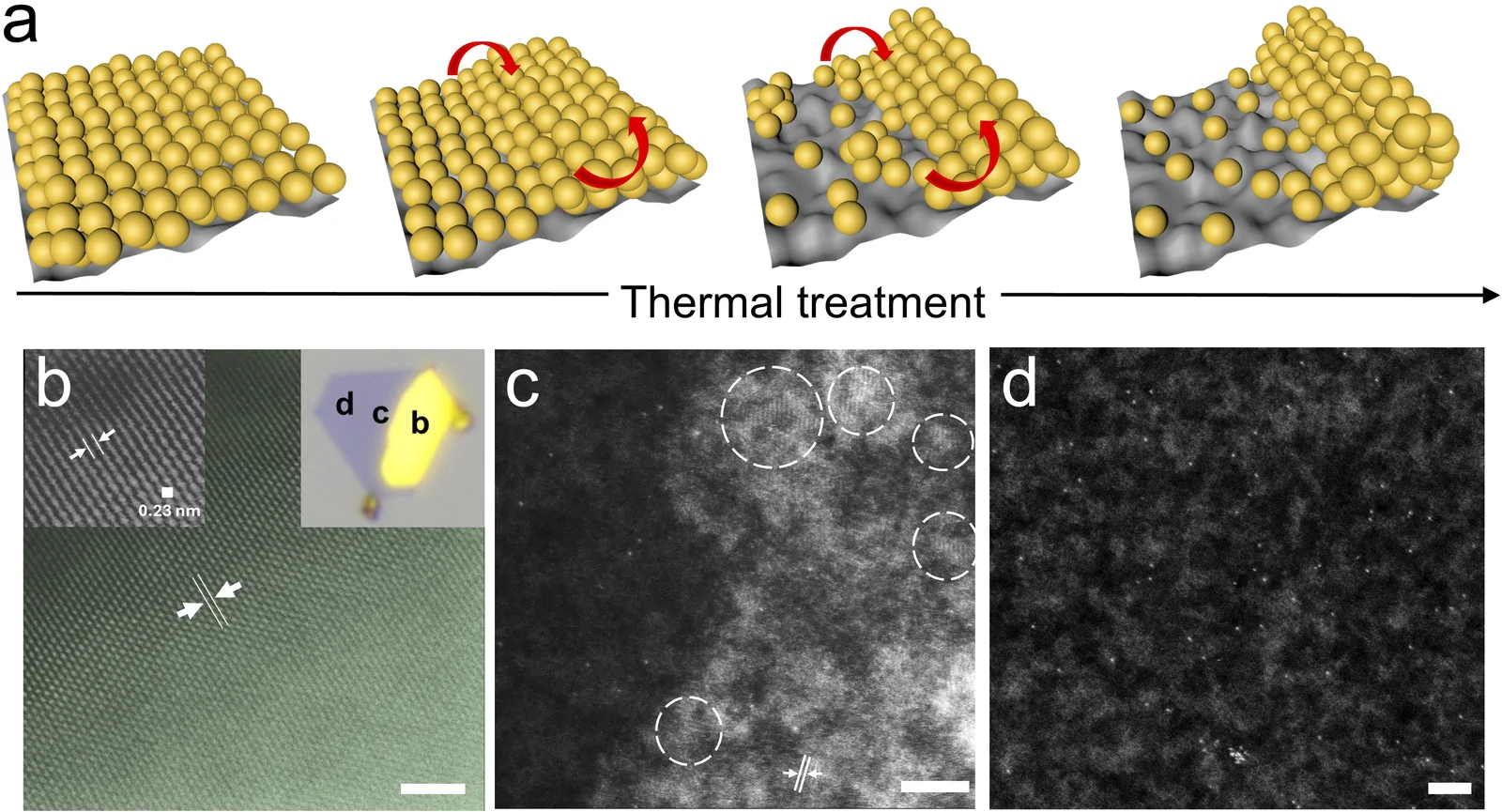
Transformation of Au nanoplates into AD-Au. (a) Illustration of the evolution process from Au nanoplates to bulk gold and AD-Au. (b) (top right inset) depicts the optical image of the annealed Au nanoplate indicating a region of aggregated bulk gold (b), an interfacial region between aggregated bulk gold and AD-Au (c), and a region of only the AD-Au on a carbonaceous support (d). (b–d) HAADF-STEM images depicting the evolution of the Au nanoplate into AD-Au. (b) Agglomerated bulk gold region from the Au nanoplate. Scale bars are 3 nm. (c) Interface region between coalesced bulk gold and AD-Au. The highlighted areas show few-nanometre large graphitic domains formed during the pyrolytic step. (d) AD-Au region on a carbonaceous support.

We propose that the carbonaceous layer originates from the organic phase of ToABr, used during the synthesis through pyrolytic reactions at high temperatures.^48,49^ It is well-known that subjecting hydrocarbon-based organic compounds to high temperatures and a chemically reducing atmosphere generally leads to their graphitization and, thus, the formation of small graphitic islands that are distributed along a primarily amorphous carbon matrix.^50^ The supporting layer is mainly composed of aliphatic carbon with sp^2^ domains to some extent and traces of oxygen-containing groups as evidenced by the C 1s line in XPS (SI, Fig. S8). After the formation of the amorphous carbon support, at temperatures exceeding 750 °C, the fast surface diffusion favours structural rearrangement of the gold nanoplate. To lower the free energy of the system, the gold plates then coalesce into shapes with a lower surface to volume ratio. During this process, some of the Au atoms become attached or embedded into the carbon-based matrix, potentially through π-stacking onto the graphitic carbon domains,^51^ which are highlighted by dashed white circles in Fig. 3c. Notably, the crystallographic orientation of the nanoplatelets is maintained when their shape is transformed into bulk gold (〈111〉 is normal to the substrate) (Fig. 3b).

The insights gathered here into the AD-Au phase mechanism of formation have been based on the state of the samples in the final stage of the relatively long annealing process. It is worth mentioning that we do not discard the possibility that in the very early stages of the transformation the mechanism could be more complex, especially regarding the carbonaceous layer formation from the residual precursor entrapped in the metallic phase. Its migration to the interface between the nanoplate and substrate could affect the overall dynamics of the process. In stages not much longer than that, which extends throughout the long hours of the thermal treatment, surface diffusion over the carbonaceous layer dominates thus defining the solid-state SSD as the major mechanism. Unfortunately, the exact mechanism taking place earlier in the process as well as the dynamics involving the carbonaceous layer formation cannot be directly probed. Moreover, during such a long stage, we do not dismiss the fact that single adatom diffusion could be disrupted by local agglomeration to a minor extent, for instance, driven by Ostwald ripening.^52^ This could explain the slightly larger and brighter motifs in the HAADF-STEM.

In conclusion, our study presents formation of AD-Au single atoms, which originate during the thermal treatment of Au platelets. This versatile method provides a high yield of Au single atoms uniformly distributed on the carbonaceous layer and is independent of the substrate composition and geometry. A partially graphitised carbonaceous layer is formed during the thermal treatment and acts as a stable buffer between the AD-Au and the substrate of choice. Through a systematic analysis of the morphology and local structure at different stages of the process, we show that the formation of AD-Au is driven by the well-known coalescing phenomenon that takes place for metallic particles at sufficiently high temperatures. It is a simple method for achieving AD-Au which can be useful for a variety of applications such as biomedical, extended catalytic processes, and sensing.

## Methods

### Preparation of the precursor

Hydrogen tetrachloroaurate(iii) hydrate (HAuCl_4_), tetraoctylammonium bromide (ToABr), and toluene (Sigma Aldrich) were used without further purification. MiliQ water was used throughout the process. We used 25 mM HAuCl_4_ solution in water and 50 mM ToABr solution in toluene. The precursor was formulated *via* a phase transfer method, involving the migration of (AuCl_4_)^–^ ions from the aqueous solution to toluene using ToABr as a phase transfer agent. The Au to ToABr ratio was maintained at 1 : 5, where 1.6 mL of the Au solution and 4 mL of ToABr were combined and stirred for 1 minute, after which they were set aside. Once the aqueous and organic layers had distinctly separated, the upper orangish organic layer (the precursor) was carefully collected.

### Synthesis of Au nanoplates on a Si/SiO_2_ substrate

A Si substrate with a SiO_2_ (290 nm) passivation layer was used as a substrate in this work. To enhance their wettability and remove strongly bound contaminants, such as hydrocarbons, we soak the substrates in isopropyl alcohol (IPA) overnight. The substrates are subjected to a further cleaning process using sequential sonication in acetone and IPA, each for a duration of 5 minutes. They are then dried using nitrogen gas before the precursor is carefully drop-cast onto the substrate. The solution on the substrate is allowed to air dry and is then placed on a hot plate at 250 °C for 3 minutes. To complete the preparation, the substrates are thoroughly washed with toluene, acetone, and isopropyl alcohol (IPA) and once again dried using nitrogen (N_2_) gas.

### Synthesis of Au nanoplates on epitaxial graphene grown on a SiC substrate

For this study, on-axis oriented single crystalline single-side polished 6H-SiC n-type (Si crystal GmbH) substrates were used. To eliminate any polishing scratches, the substrates are subjected to hydrogen gas (H_2_) etching performed at 1210 °C for 4 min in a mixture of argon (Ar) and H_2_ atmosphere at 450 mbar pressure. The Si-terminated SiC *i.e.* SiC (0001) surfaces were then subjected to graphitization through annealing in an argon atmosphere at 750 mbar.^53^ AFM characterization is used to analyse the morphology and uniformity of the substrate (SI, Fig. S10) before the growth of Au nanoplates. The following steps are the same as those used for the Si/SiO_2_ substrate described above.

### Pyrolytic formation of AD-Au on SiO_2_ and epitaxial graphene grown on the SiC substrate for STM analysis

The as-prepared substrates carrying the Au nanoplates are put in a quartz boat and placed in a tube furnace. The samples are heated to 750 °C with a heating rate of 5 °C min^−1^ and kept for 12 hours under the flow of an Ar-10% H_2_ atmosphere and are then naturally cooled to room temperature while still in the furnace.

### Wet transfer method to prepare TEM samples

We used the wet-transfer method, which is routinely used for transferring 2D material flakes, to lift the Au-nanoplate off the Si/SiO_2_ substrate and transfer it onto the TEM grid. Initially, the Si/SiO_2_ substrate, carrying the desired Au-nanoplate, is spin-coated with polymethyl methacrylate (PMMA), 950 K molecular weight, 3% (wt/vol) in anisole solvent. Once dried, the wafer is immersed in a dilute KOH solution (approximately 18% wt/vol) to gradually etch away the silicon oxide until the PMMA membrane, carrying the targeted Au-nanoplate along with its carbonaceous supporting layer, floats in the solution. This process typically takes between 6 and 10 hours. The floating PMMA membrane is then carefully washed by immersion in deionized water, after which the Au-nanoplate is ready for transfer onto the target substrate, in this case, the TEM grid. It is important to note that one can accelerate the KOH etching process by using a higher-concentration KOH solution at an elevated temperature. However, to mitigate the risk of contamination from high-concentration KOH and the potential melting of tape, we use dilute KOH solutions at room temperature. Here, an in-house built transfer rig is used to perform the transfer process. The Au-nanoplate, along with the PMMA membrane, is attached to double-sided sticky tape on a plectrum, which allows independent motion in the *x*, *y*, and *z* directions. The target substrate is secured through vacuum suction on a temperature-controlled stage. Once the Au-nanoplate and target substrate are precisely aligned, the transfer arm with the plectrum is lowered until the target substrate is in proper contact with the PMMA membrane. Adjusting the temperature of the heating stage aids in controlling the adhesion between the Au-nanoplate and the substrate, ensuring a successful transfer.

### XPS analysis

X-ray Photoelectron Spectroscopy (XPS) measurements were carried out using an Axis Supra spectrometer manufactured by Kratos Analytical in the United Kingdom. This instrument features a monochromatic Al-Kα X-ray source operating at 30 mA and 15 kV, while maintaining a base vacuum pressure of approximately 5 × 10^−9^ mbar. A spot-size of 300 × 700 µm^2^ was employed to ensure that all surveys and high-resolution individual lines accurately represented the average structure and electronic characteristics of the sample. Neutralization adjustments were made using a coupled filament, and the binding energy scale was calibrated based on the C–C band observed in the C 1s photoelectron peak at 285 eV. Besides the overall survey, high-resolution scans were obtained for both Au 4f (bulk Au on the Si/SiO_2_ substrate and AD-Au) and C 1s (from the amorphous carbon-based supporting layer) lines. A bulk Au sample, prepared *via* thermal evaporation on Si/SiO_2_, served as the reference for detecting and analysing changes in the Au spectral lines. All spectra were processed using standard Casa XPS software,^54^ with Shirley background fitting and Gaussian and asymmetric pseudo-Voigt functions for the high-resolution Au 4f and C 1s^55^ peaks, respectively.

To extract the Auger parameters, XPS data were acquired using a Kratos Axis SUPRA using monochromated Ag Lα (2984.3 eV) X-rays at 15 mA emission and 12 kV HT (180 W) and a spot size/analysis area of 700 × 300 µm. The instrument was calibrated to gold metal Au 4f (83.95 eV) and dispersion was adjusted to result in a BE of 932.6 eV for the Cu 2p_3/2_ line of metallic copper. Ag 3d_5/2_ line FWHM at 10 eV pass energy was 0.544 eV. Source resolution for monochromatic Ag Lα X-rays is ∼0.5 eV. High resolution spectra were obtained using a pass energy of 40 eV, step size of 0.1 eV and sweep time of 60 s, resulting in a peak width of 1.05 eV for Au 4f_7/2_. Survey spectra were obtained using a pass energy of 160 eV. All data were recorded at a base pressure of below 9 × 10^−9^ torr and at room temperature (294 K). Data were analysed using CasaXPS v2.3.19PR1.0. Peaks were fit with a Shirley background prior to component analysis. MNN Auger data were smoothed prior to plotting for ease of view.

### Raman spectroscopy

Raman spectra were acquired utilizing a Renishaw inVia micro-spectrometer equipped with a 532 nm excitation laser and a 50× objective. To prevent any potential damage to the samples caused by either local heating, or ablation, or even ejection of the single atoms, the measurements were conducted at 50% of the laser's maximum power (50 mW). Multiple spectra were recorded to ensure the statistical reliability of the data and to obtain an averaged representation of the sample's structural information.

### Scanning tunnelling microscopy

The scanning tunnelling microscopy (STM) and scanning tunnelling spectroscopy (STS) analysis was performed with a variable-temperature (VT) ultra-high-vacuum STM from RHK Technology with a base pressure of 3 × 10^−11^ mbar. The scanning tips, derived from a tungsten wire with a diameter of 250 µm, were prepared through electrochemical etching, *in situ* degassing, and flashing *via* the application of high voltage before usage. *In situ* degassing of the samples was achieved by flowing a DC current through the substrate. The annealing temperature, monitored using an infrared pyrometer and a thermocouple, was maintained at either 300 °C or 450 °C overnight. The STM images were recorded in constant-current mode using several values for bias and current. The images were analysed with the WSXM software package.^56^

### Transmission electron microscopy

The atomic structure, Au atom distribution and chemical composition of AD-Au were assessed by scanning transmission electron microscopy (STEM) and energy dispersive X-ray spectroscopy (EDS) using a Thermo Fisher Titan STEM (G2 80-200) operating at 200kV, and equipped with a Cs probe corrector (CEOS) and a high-angle annular dark-field (HAADF) detector. HAADF STEM images were recorded with a probe convergence angle of 18 mrad and an inner collection angle of 55 mrad.

## Conflicts of interest

There are no conflicts to declare.

## Supplementary Material

NA-008-D6NA00466K-s001

## Data Availability

Source data presented in the figures are provided here: https://figshare.com/s/b41e30bf65ff30727fda. Supplementary information (SI): additional morphological characterisation from optical and transmission electron microscopy, XPS assessment of both carbonaceous layer and Au MNN Auger parameter, and STM analysis. See DOI: https://doi.org/10.1039/d6na00466k.
