# Supplementary material for: Solid-state dewetting templates the formation of an atomically dispersed gold phase
Source: Nanoscale Adv. 2026 Jun 25. Online ahead of print. doi: 10.1039/d6na00466k (PMC13390749; doi:10.1039/d6na00466k)
Supplement: NA-OLF-D6NA00466K-s001 [file NA-OLF-D6NA00466K-s001.pdf]

Supplementary Information (SI) for Nanoscale Advances.
This journal is © The Royal Society of Chemistry 2026

Supplementary Information

Solid-state dewetting templates the formation of an atomically dispersed gold phase

Ravalika Sajja,[a,b] Marcos Vinicius Surmani Martins,[a,b] Rongsheng Cai,[c] Abdulghani Ismail,[a,b] Gwang-Hyeon Nam,[a,b] Ylea Vlamidis,[d,e] Neeraj Mishra,[f,g] Max Rimmer,[a,b] Stefan Heun,[d] Camilla Coletti,[f,g] Mark A. Isaacs,[h,i] Ashok Keerthi,[j] Sarah J. Haigh,[c] Stefano Veronesi,[d] Boya Radha[a,b†]

a) *Department of Physics & Astronomy, The University of Manchester, Oxford Road, Manchester M13 9PL, United Kingdom*
b) *National Graphene Institute, The University of Manchester, Oxford Road, Manchester M13 9PL, United Kingdom*
c) *Department of Materials, The University of Manchester, Oxford Road, Manchester M13 9PL, United Kingdom*
d) *Istituto Nanoscienze – CNR, NEST – Scuola Normale Superiore, Piazza S. Silvestro 12, 56127 Pisa, Italy*
e) *Department of Physical Science, Earth, and Environment, University of Siena, Via Roma 56, 53100, Siena, Italy*
f) *Center for Nanotechnology Innovation @ NEST, Istituto Italiano di Tecnologia, Piazza S. Silvestro 12, 56127 Pisa, Italy*
g) *Graphene Labs, Istituto Italiano di Tecnologia, Via Morego 30, Genova 16163, Italy*
h) *Department of Chemistry, University College London, 20 Gordon Street, London WC1H 0AJ, United Kingdom*
i) *HarwellXPS, Research Complex at Harwell, Rutherford Appleton Labs, Harwell Campus OX11 0FA, United Kingdom*
j) *Department of Chemistry, The University of Manchester, Oxford Road, Manchester M13 9PL, United Kingdom*

[†] Corresponding author: B.R. (radha.boya@manchester.ac.uk)

1) **Synthesis of Au nanoplates on Si/SiO_2 substrate:** Optical images of the as-prepared Au microplates, reveal the simultaneous formation of multiple plates in a single instance. This allows for efficient and high-yield production with scalability, appealing for various applications.

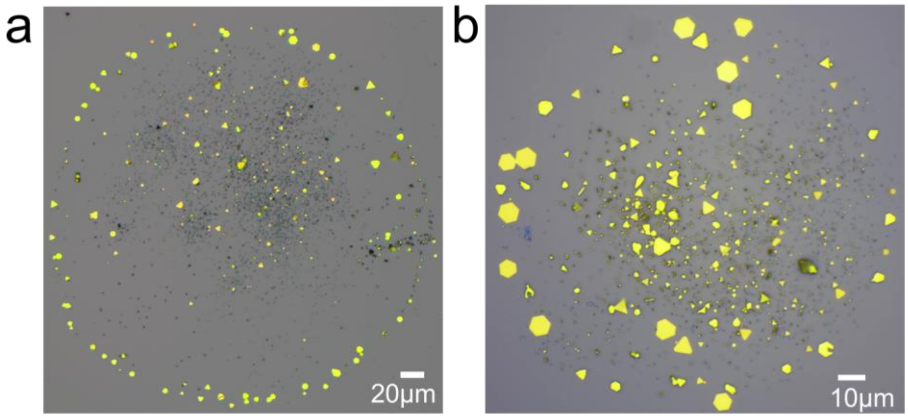

Figure S1 | Typical optical images of the as-prepared Au microplates. Multiple nanoplate formation occurs simultaneously. The images in a and b are from different samples. The contours of the drop-casted droplets are visible from the patterns formed along with the freshly grown micro-/nanoplates.

2) **Formation of AD-Au on different substrates:** We demonstrate the adaptability of our synthesis method when applied to different substrates, such as quartz (a, b), epitaxial graphene on SiC (c, d) is demonstrated. This method can be readily adapted and tailored to different materials if they are able to sustain the pyrolysis temperature, emphasizing the versatility of our synthesis method and potential for a wide application.

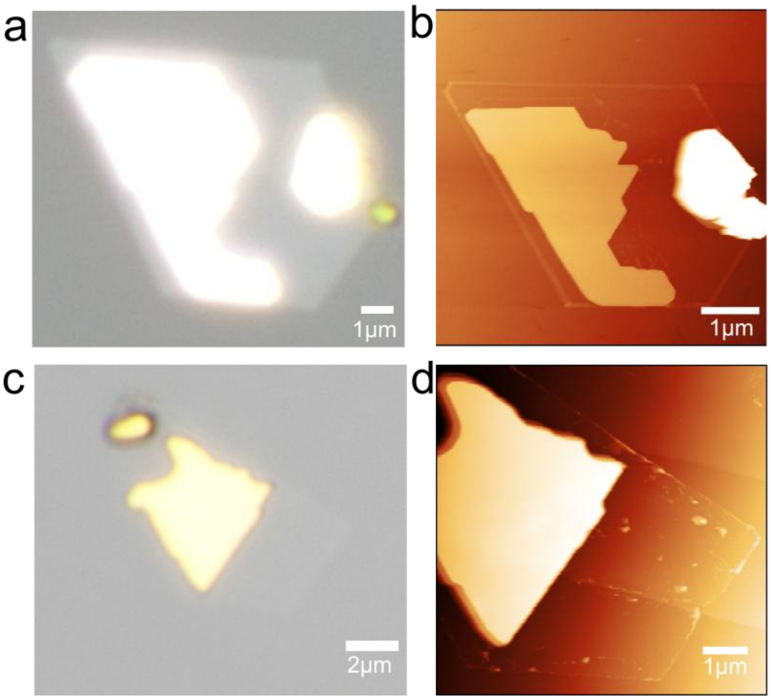

Figure S2| Versatility of our synthesis method for various other substrates (a,c) Optical images and (b,d) AFM images of annealed Au nanoplate on quartz substrate and on epitaxial graphene on SiC substrate, respectively.

3) **Influence of the atmosphere used in the pyrolytic process:** We varied the gas environment during the pyrolysis for generation of AD-Au to study its influence on the process. The pyrolysis reaction is investigated under Ar/H_2, high-vacuum, and oxygen conditions. We observe the formation of the amorphous carbon matrix only for pyrolysis under the highly reducing Ar/H_2 flow. Under vacuum conditions, only residues of the matrix are identified, which is likely due to sublimation, and a clean substrate is observed under oxygen flow as also verified by AFM (Figure S3 d-f). Raman results further confirm the absence of any carbon vibration modes (Figure S3 g-i). The vacuum annealing poorly results in the formation of the amorphous carbon film, and oxygen (or oxidising, in general) triggers the combustion of the carbon source beneath the Au nanoplate. Therefore, reducing atmosphere such as Ar/H_2 is a crucial parameter for securing the yield of AD-Au along the carbonaceous matrix.

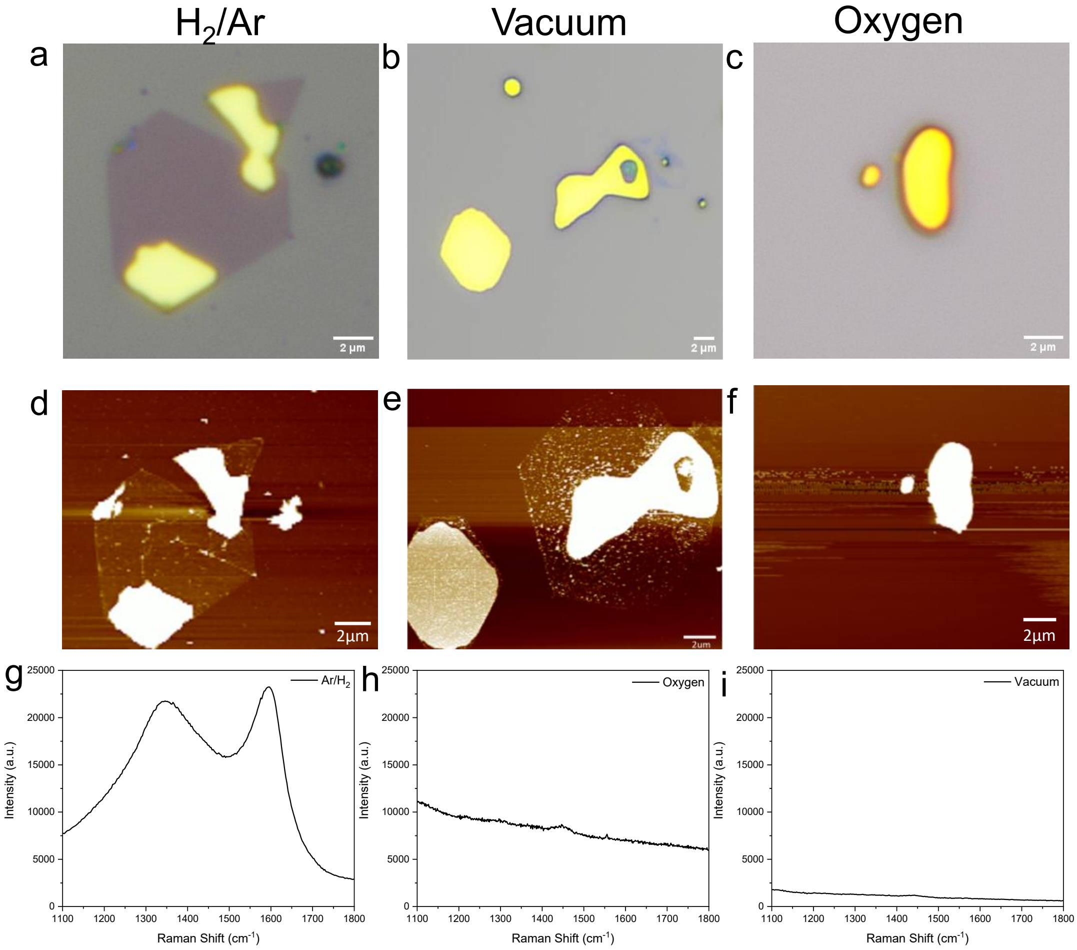

Figure S3| Influence of gas environment on the synthesis of AD-Au. (a-c) Optical images, (d-f) AFM images and (g-i) Raman spectra of Au nanoplates upon annealing in Ar/H_2 (same as shown in figure 2h), vacuum, and oxygen, respectively. Raman spectra from the carbonaceous supporting layer show the presence of amorphous carbon peaks only for the samples annealed in Ar/H_2 environment – the graphic scales were maintained the same for a reliable comparison.

4) **AFM microscopy on coalesced bulk Au:** AFM micrographs provide additional evidence supporting the mechanism underlying the thinning of Au nanoplates and the subsequent gradual coalescence that leads to the formation of bulk Au. An analysis of different areas of the coalesced bulk nanoplate supports our proposed mechanism. The AFM profiles presented below clearly depict a gradual increase in the height of the aggregated bulk gold moved away from the thin Au plate. Notably, the region closer to the interface (cyan profile) appears flatter, whereas the region farther from the interface (blue profile) appears non-flat, suggesting the migration and aggregation of Au atoms.

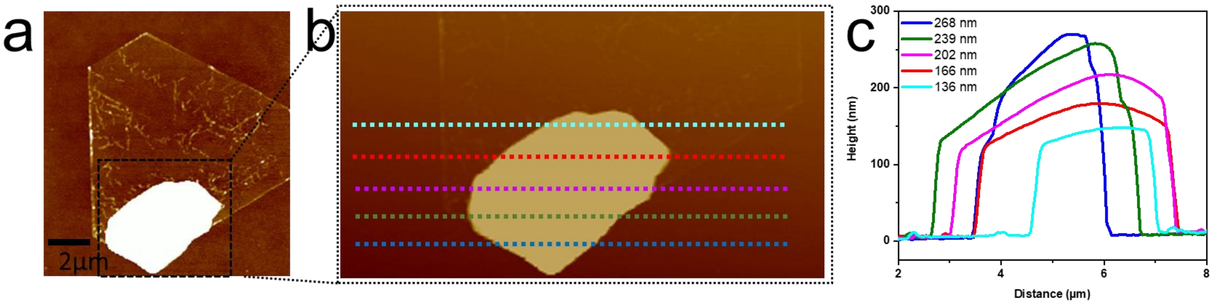

Figure S4| (a) AFM image capturing the entire nanoplate including the aggregated bulk gold region and the AD-Au region (reproduced image from figure 1e in main, for comparison), (b) Zoomed in AFM image focusing on the aggregated bulk gold region, highlighting the location of the line profiles. (c) Line profile corresponding to the regions identified in panel (b), providing information about the height and morphology of the aggregated bulk gold.

5) **Dense distribution of Au atoms:** We provide here additional images of HAADF-STEM analysis acquired from various regions within the synthesized AD-Au nanoplate. From the multiple regions, we can see uniform and widespread dispersion of Au-SAs.

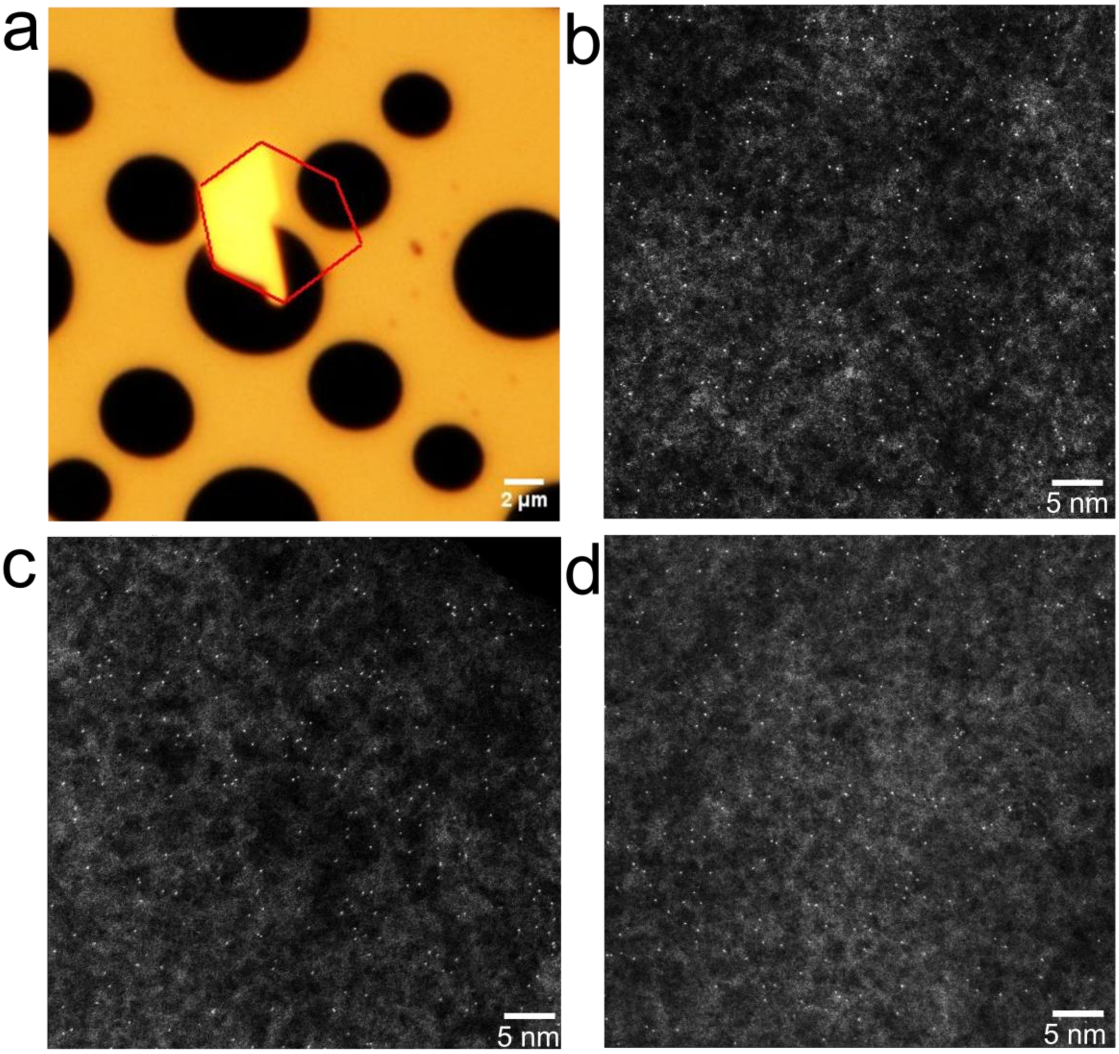

Figure S5| (a) Optical image of an annealed Au nanoplate onto a TEM grid. (b-d) HAADF-STEM images demonstrating atomically dispersed gold attained from different regions of AD-Au nanoplate. The Au atoms are uniformly and densely dispersed on a carbonaceous matrix.

6) Auger parameter analysis:

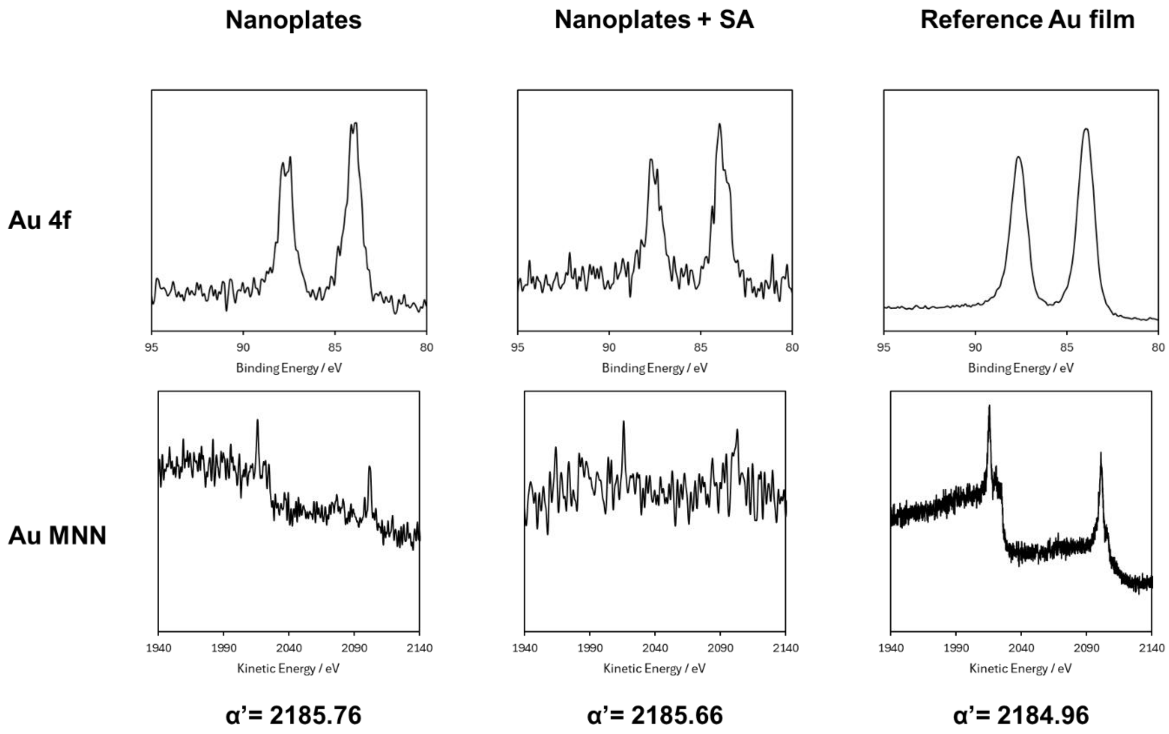

Figure S6| Auger parameter variation across the AD-Au formation process. Au 4f (top panels) and Au MNN (lower panels) for nanoplates, nanoplates+single atoms (SA), and reference Au film collected with Ag L-α X-ray source.

7) **Electron diffraction (ED) over the AD-Au region:** The presence of a diffuse ring in the SAED pattern is a direct indication of highly amorphous carbonaceous region supporting the AD-Au phase.

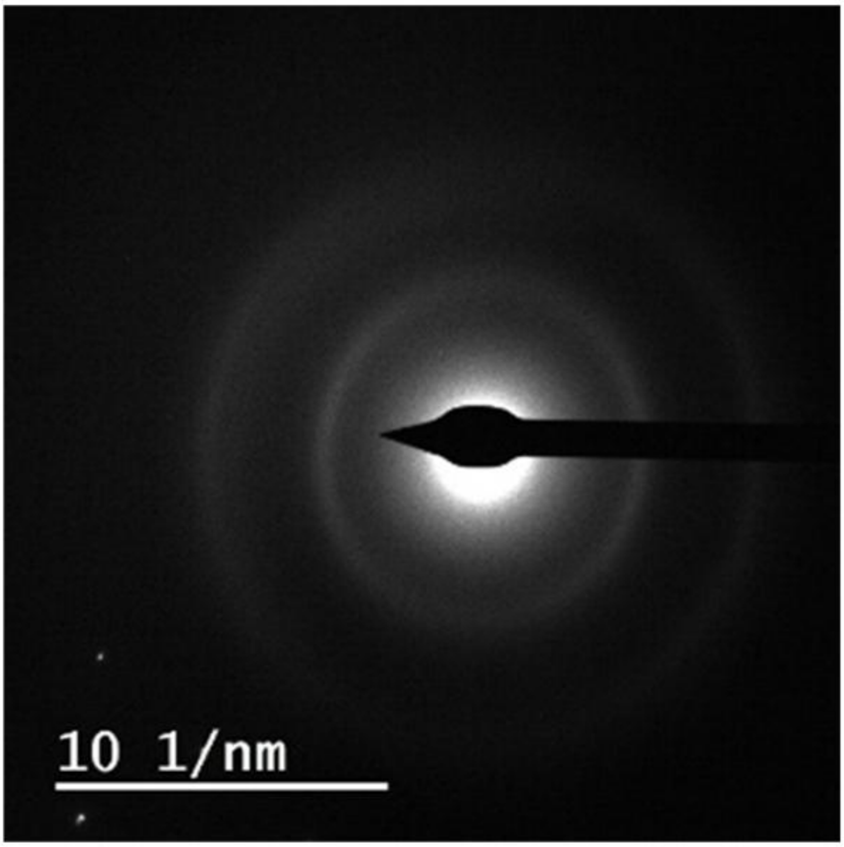

Figure S7| Sample diffused ring from SAED pattern obtained from the carbonaceous support.

8) **Compositional signature of the carbonaceous layer:** Anchoring of the Au structures can also be influenced by the structure and composition of the substrate. To explore that further, we have looked at the C 1s line, which gives direct information on the structure and composition of the C-based layer. The spectra indicate that this layer is mainly composed by aliphatic carbon, which is expected to be highly amorphous when originated from pyrolytic reactions such as our case. Small amounts of sp^2 carbon and oxygen-containing groups were also detected, supporting our STEM-EDS data. The density of C-sp^2 domains varies from sample-to-sample. The C-O bonds could be generated from mild secondary reactions during the pyrolysis between the carbonaceous layer being formed and the SiO_2 layer under reducing atmosphere. Nonetheless, C-sp^3 and C-sp^2 are the predominant and consistent across the samples.

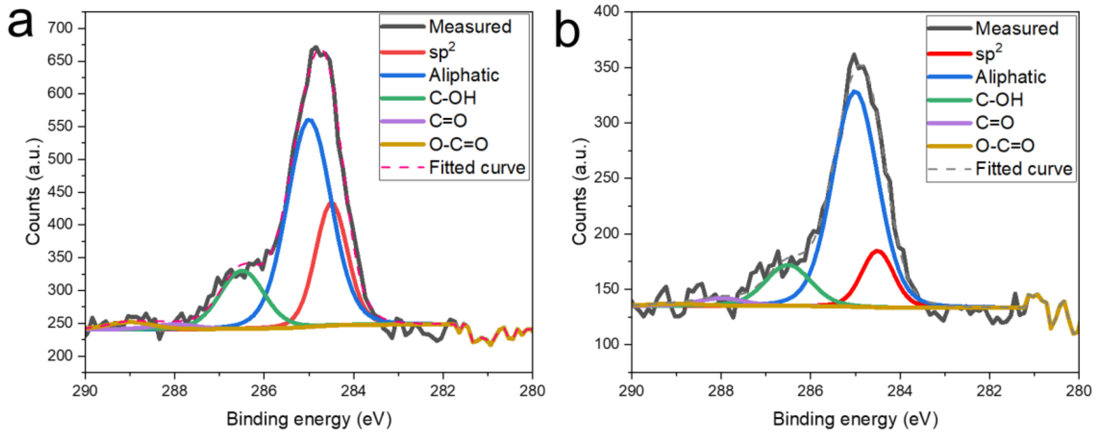

Figure S8| XPS C 1s fingerprint from the carbonaceous layer supporting the AD-Au (a, b) Sample spectra of the series of analysed samples.

9) Scanning tunnelling microscopy (STM) and scanning tunnelling spectroscopy (STS):

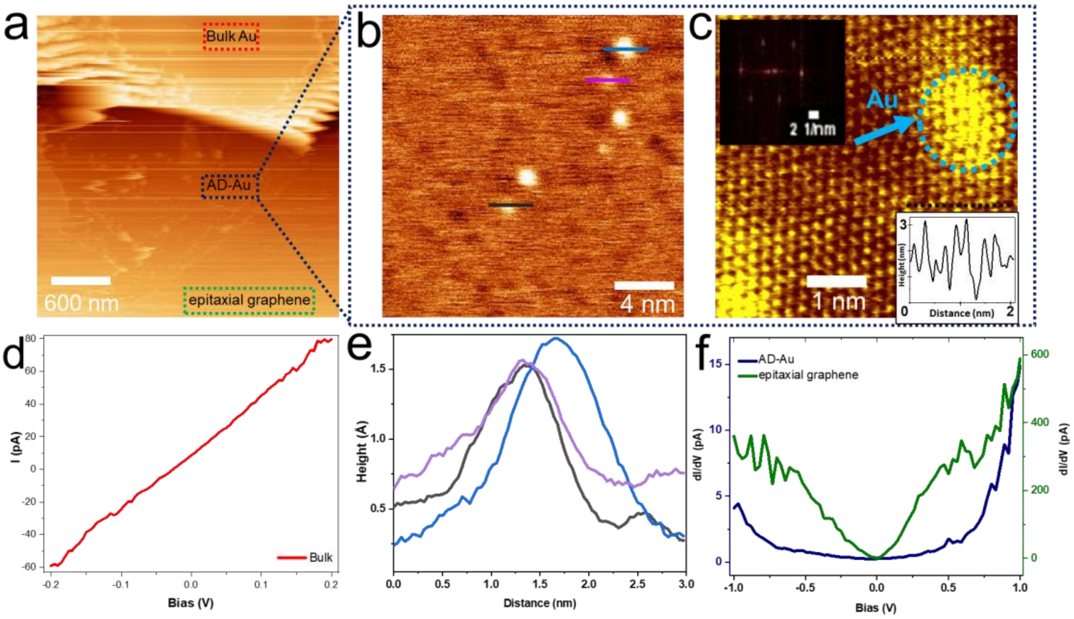

Figure S9| Characterisation of AD-Au using STM and STS. (a) Scanning tunnelling microscope (STM) image of annealed Au nanoplates indicating the agglomerated bulk region, AD-Au region and the epitaxial graphene, grown on 6H-SiC substrate. (b) Zoomed in region (from the marked region, not to scale) of AD-Au indicating bright dots that have a diameter corresponding to individual atomic species or few atom aggregates, the profiles of these are shown in (e). (c) Atomically resolved STM image showing epitaxial graphene in the AD-Au region. Line profile (right bottom inset) showing the expected lattice spacing of the graphene lattice (~0.24nm). The fast Fourier transform (left bottom inset) shows graphene's characteristic hexagonal lattice, (d, f) Scanning tunnelling spectroscopy of the agglomerated bulk gold, AD-Au and epitaxial graphene on the SiC substrate. (d) Current (I) versus the sample bias voltage (V) spectra obtained on the bulk gold indicates metallic behaviour. (f) Differential conductivity (dI/dV) versus the sample bias voltage (V) obtained on AD-Au region indicates semiconductor behaviour and on the epitaxial graphene outside AD-Au area indicates semi-metal behaviour.

10) AFM characterisation of the epitaxial graphene on SiC substrate: AFM micrographs on the epitaxial graphene indicating the morphology of the substrate before the synthesis of the Au nanoplates.

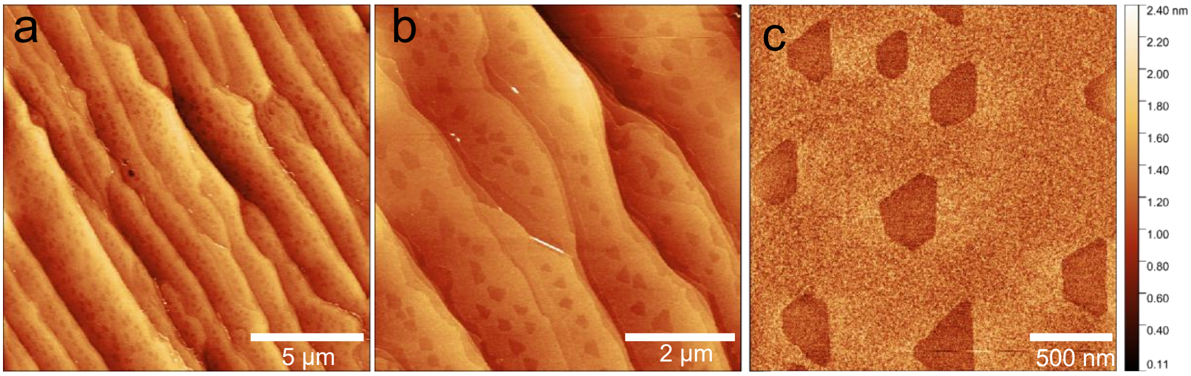

Figure S10| AFM micrographs of bare epitaxial graphene grown on SiC substrate. (b) AFM micrograph of the SiC substrate morphology with step-like features (c) High magnification AFM images showing islands of bilayer graphene.
